# Inhibition of Amebic Lysosomal Acidification Blocks Amebic Trogocytosis and Cell Killing

**DOI:** 10.1128/mBio.01187-17

**Published:** 2017-08-29

**Authors:** Allissia A. Gilmartin, Katherine S. Ralston, William A. Petri

**Affiliations:** a Department of Microbiology, Immunology and Cancer Biology, University of Virginia, Charlottesville, Virginia, USA; b Department of Microbiology and Molecular Genetics, University of California, Davis, California, USA; c Department of Medicine, University of Virginia, Charlottesville, Virginia, USA; d Department of Pathology, University of Virginia, Charlottesville, Virginia, USA; University of California, Los Angeles

**Keywords:** *Entamoeba histolytica*, enteric pathogens, host-pathogen interactions, trogocytosis

## Abstract

*Entamoeba histolytica* ingests fragments of live host cells in a nibbling-like process termed amebic trogocytosis. Amebic trogocytosis is required for cell killing and contributes to tissue invasion, which is a hallmark of invasive amebic colitis. Work done prior to the discovery of amebic trogocytosis showed that acid vesicles are required for amebic cytotoxicity. In the present study, we show that acidified lysosomes are required for amebic trogocytosis and cell killing. Interference with lysosome acidification using ammonium chloride, a weak base, or concanamycin A, a vacuolar H^+^ ATPase inhibitor, decreased amebic trogocytosis and amebic cytotoxicity. Our data suggest that the inhibitors do not impair the ingestion of an initial fragment but rather block continued trogocytosis and the ingestion of multiple fragments. The acidification inhibitors also decreased phagocytosis, but not fluid-phase endocytosis. These data suggest that amebic lysosomes play a crucial role in amebic trogocytosis, phagocytosis, and cell killing.

## IMPORTANCE

*E. histolytica* is a protozoan parasite that is prevalent in low-income countries, where it causes potentially fatal diarrhea, dysentery, and liver abscesses. Tissue destruction is a hallmark of invasive *E. histolytica* infection. The parasite is highly cytotoxic to a wide range of human cells, and parasite cytotoxic activity is likely to drive tissue destruction. *E. histolytica* is able to kill human cells through amebic trogocytosis. This process also contributes to tissue invasion. Trogocytosis has been observed in other organisms; however, little is known about the mechanism in any system. We show that interference with lysosomal acidification impairs amebic trogocytosis, phagocytosis, and cell killing, indicating that amebic lysosomes are critically important for these processes.

## INTRODUCTION

*Entamoeba histolytica* is a protozoan parasite that is prevalent in low-income countries. In humans, the parasite causes potentially fatal invasive colitis, which is seen in 10 to 25% of patients, and extraintestinal abscesses, which occur in about 1% of patients (1, 2). Worldwide, diarrheal disease is the second leading cause of death for children under 5 years old (3). In an urban slum of Dhaka, Bangladesh, we found that 80% of children were infected with *E. histolytica* at least once over a 4-year period and 53% experienced repeated infections (4). Repeated infections in children are particularly serious as they are associated with chronic malnourishment, stunting, and cognitive defects (5).

Tissue destruction is the hallmark of invasive *E. histolytica* infection, manifesting as massive intestinal ulceration or abscesses in other sites. *E. histolytica* is highly cytotoxic to a wide range of human cells, and the parasite’s cytotoxic activity is likely to drive tissue destruction. It was recently discovered that *E. histolytica* kills by ingesting fragments of live host cells, which has been termed amebic trogocytosis (6). This process begins with attachment of the parasite to the host cell, which is mediated in large part by the parasite’s Gal/GalNAc lectin (6–8). Following attachment, the parasites ingest fragments of the host cell. These fragments were shown to contain host cell membrane, cytoplasm, and mitochondria. The parasites continue ingesting fragments of the host cell until the host cell eventually dies. Notably, it has been demonstrated that while amebic trogocytosis initiated rapidly, host cell death did not occur until several minutes later, after the amebae had ingested multiple fragments. The number of ingested fragments is likely critical for eliciting host cell death, since pharmacological and genetic inhibitors that quantitatively reduced the number of ingested fragments almost completely inhibited host cell death (6). These data suggest that cell death results after a threshold of physical damage has been crossed. However, we currently lack an understanding of the mechanism that underlies amebic trogocytosis and cell killing.

A process morphologically similar to amebic trogocytosis has been observed in other organisms. Human lymphocytes, including T, B, natural killer (NK), and dendritic cells and macrophages undergo a process that has also been called trogocytosis (9). In lymphocytes, trogocytosis has been implicated in cell-cell communication (9). The process is distinguished from other methods of intracellular transfer, such as phagocytosis, by the transfer of fragments of cell material (including intact proteins but not whole cells), the requirement for close cell-cell contact, and the high rate of uptake (within minutes), all of which are reminiscent of amebic trogocytosis (9). In T and NK cells, trogocytosis is a metabolically active process that requires signaling in the acceptor cell and modulation of both the actin cytoskeleton and intracellular Ca^2+^ (minireview in reference 10). The small GTPases TC21 and RhoG and phosphatidylinositol 3-kinase (PI3K), were identified as key players in T-cell trogocytosis (11). A process referred to as trogocytosis also been observed in the free-living ameba *Naegleria fowleri*, a human parasite that may use this process to destroy host cells, but again the mechanism is unknown (12–14). Finally, a trogocytosis-like process observed in *Plasmodium falciparum*-infected red blood cells has been implicated in the pathogenesis of cerebral malaria (15).

Previous work demonstrated that amebic trogocytosis leads to an irreversible elevation of host cell calcium, followed by death. It was shown that increasing levels of amebic trogocytosis correspond with increasing levels of host cell death. Moreover, inhibition of amebic trogocytosis using a range of methods, including PI3K inhibition and blockade of the amebic surface receptor Gal/GalNAc lectin, resulted in a concomitant decrease in cell killing. These data indicate that amebic trogocytosis results in cell killing (6). It has been observed that host cell death occurs after sustained ingestion of many fragments (6). Importantly, the numerous ingested cell fragments acquired during amebic trogocytosis must be processed. In eukaryotes, lysosomes are critical for the degradation of ingested macromolecules. Lysosomal function is therefore likely to be essential to support continued trogocytosis and, consequently, host cell killing. It has been demonstrated that elevated amebic lysosomal pH decreases cytotoxicity, suggesting that functional lysosomes are required for cell killing (16). In addition, work on EhRab7B, an amebic *E. histolytica* Rab GTPase that is implicated in lysosomal maturation and late endosome/lysosome fusion, has shown that interference with EhRab7B results in decreased phagocytosis, phagosome acidification, and degradation of ingested cells (17). These findings suggest that there is a crucial role for the degradation pathway in cell killing. Interestingly, previous work also found that phagosomes in *E. histolytica* were acidified much more quickly and reached a lower pH than phagosomes in the less-virulent organism *Entamoeba dispar*, suggesting that phagosome acidification, and therefore amebic lysosomes, may play a role in amebic virulence (18).

Based on these findings, we hypothesized that amebic lysosomes are essential for continued amebic trogocytosis and host cell killing. In this study, we examined the impact of impaired lysosomal acidification on amebic trogocytosis and cell killing. Using imaging flow cytometry to quantitatively assess the rates of trogocytosis and host cell killing, we found that inhibition of lysosomal acidification significantly decreased amebic trogocytosis, phagocytosis, and cell killing, indicating a crucial role for amebic lysosomes in these processes. This work sheds new light on an observation, first made 30 years ago, that weak bases inhibit amebic killing of human cells, by demonstrating that acid vesicle neutralization acts through the inhibition of trogocytosis.

## RESULTS

### Acidification inhibitors decrease amebic trogocytosis and cell killing.

*E. histolytica* can kill human cells through amebic trogocytosis (6). Previous work has shown that elevated amebic lysosomal pH decreased cytotoxicity, suggesting that functional lysosomes are required for cell killing (16). To determine whether lysosomes are required for amebic trogocytosis and cell killing, we used two independent pharmacological inhibitors of lysosomal pH: concanamycin A and ammonium chloride. Concanamycin A is a specific inhibitor of vacuolar H^+^ ATPases (V-ATPases). V-ATPases function as proton pumps, and their inhibition has been shown to block acidification of vesicles in *E. histolytica* (19–21). In ciliated protozoa, V-ATPases are also required for targeting and subsequent tethering of the acidified vesicle to its target membrane (reviewed in reference 22). Treatment of *E. histolytica* with concanamycin A has been shown to inhibit acidification of phagosomes and degradation of ingested bacteria and yeast (18). Ammonium chloride, a weak base, also raises the pH of amebic lysosomes and was previously shown to inhibit host cell killing (16).

**FIG 1:**
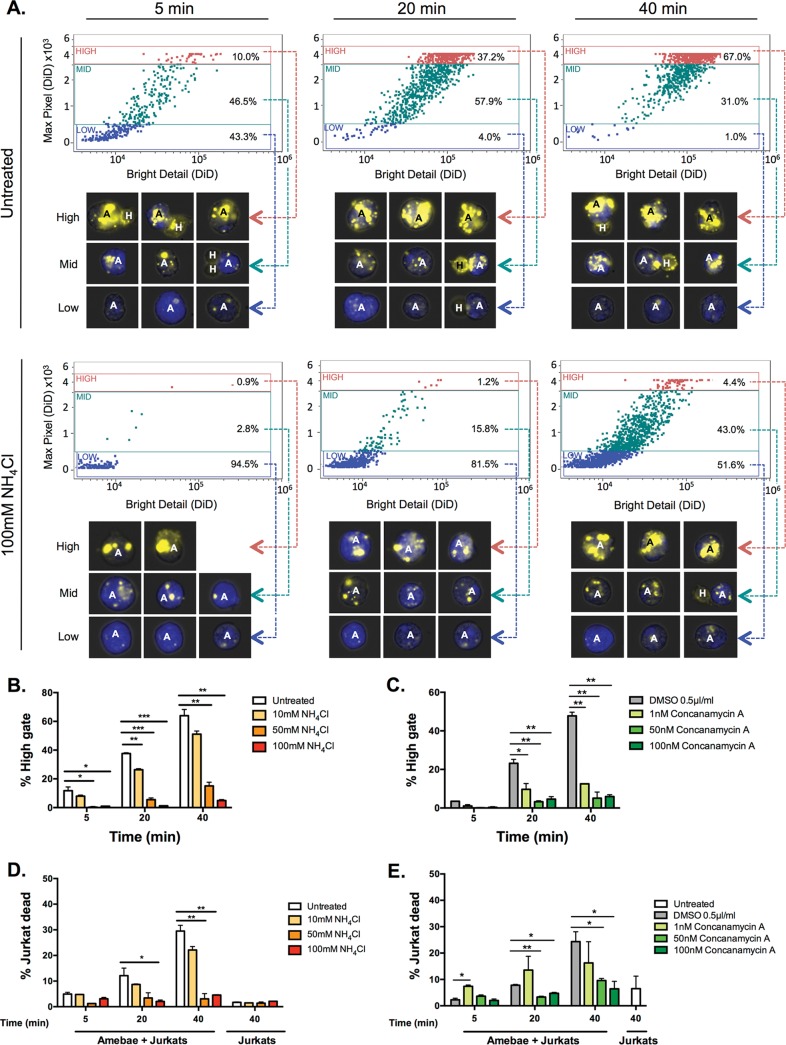
Acidification inhibitors decrease trogocytosis and cell killing. Amebae pretreated with ammonium chloride for 1 h were incubated with Jurkat cells for 5, 20, or 40 min in medium containing ammonium chloride. Amebae pretreated with concanamycin or vehicle for 1 h were washed and incubated with Jurkat cells for 5, 20, or 40 min in medium without inhibitor or vehicle. Afterward all cells were stained with LIVE/DEAD violet on ice for 30 min and then fixed. Amebic trogocytosis and cell killing were analyzed using imaging flow cytometry. (A) Measurement of fragmentation of ingested material over time. (B and C) The percentage of events in the high gate reflects the quantity of fragments that have been ingested by the parasites. (D and E) Percentage of Jurkat host cells staining with LIVE/DEAD violet. Means and standard deviations are for biological duplicates (10,000 events/each). Data were analyzed by one-way analysis of variance (ANOVA) using Prism 6. *, *P* ≤ 0.05; **, *P* ≤ 0.01; ***, *P* ≤ 0.001.

5-Chloromethylfluorescein diacetate (CMFDA)-labeled parasites were treated with either concanamycin A or ammonium chloride for 1 h. Following this incubation, the concanamycin A-treated parasites were washed extensively to remove free inhibitor and then coincubated with DiD-labeled human Jurkat T cells for 5, 20, or 40 min in medium alone. Thus, only the amebae, not the Jurkat T cells, were exposed to concanamycin A. Previous studies have shown that *E. histolytica* is able to rapidly reacidify lysosomes within minutes of ammonium chloride removal; therefore, ammonium chloride was maintained in the medium throughout the coincubation with human cells (16). After coincubation of the parasites with the human cells, all cells were stained with the permeability dye LIVE/DEAD violet and fixed. We then assessed trogocytosis and cell killing using imaging flow cytometry (Fig. 1). To quantify trogocytosis, we measured fragmentation of the human material within the amebae, as previously described by Ralston et al. (Fig. 1A; Fig. S1 in the supplemental material shows the full gating strategy).

10.1128/mBio.01187-17.1 FIG S1  Gating strategy for imaging flow cytometry. CMFDA-labeled amebae were incubated with DiD-labeled Jurkat cells. Dead cells were stained with LIVE/DEAD violet. Acquisition was performed on an Amnis ImagestreamX Mark II flow cytometer. A total of 10,000 events were collected. Analysis was performed using IDEAS software (panel 1). Focused events were selected using a bright-field gradient (panel 2). Events gated in panel 1 were enriched for single events by removing compound events containing two or more cells not in contact (panel 3). Events gated in panel 2 were plotted based on aspect ratio and intensity of CMFDA staining to distinguish between single Jurkat cells, single amebae, and clusters of amebae (panel 4). The single amebae identified in panel 3 were assessed for the presence of human material, including fragments, whole human cells, or both. Events gated as “Jurkat Positive” had some human material (panel 5). Events identified as “Jurkat Positive” were examined for internalization of the human material using the internalization score feature, which assesses the overlap between the DiD image and a mask based on the CMFDA image. Events gated as “Internalization +” had internalized some human material (panel 6). To assess how much human material was internalized and whether the material was intact or fragmented, the “Internalization +” events gated in panel 5 were examined using the bright detail and maximum pixel intensity of DiD. Events were gated as “Low,” “Mid,” or “High” based on these parameters. The three gates “Low,” “Mid,” and “High” were maintained in a constant position throughout all experiments. (Panel 3a) To assess viability of Jurkat cells, events gated as “Single Jurkats” in panel 3 were plotted based on granularity (side scatter [SSC]) and intensity of LIVE/DEAD violet. (Panel 3b) To assess viability of amebae, events gated as “Single Amebae” in panel 3 were plotted based on granularity (side scatter) and the overlap of CMFDA and LIVE/DEAD violet. Download FIG S1, TIF file, 2.6 MB. Copyright © 2017 Gilmartin et al. 2017 Gilmartin et al. This content is distributed under the terms of the Creative Commons Attribution 4.0 International license.

In untreated amebae, the percentage of amebae that ingested a high number of human fragments increased over time from 10.0% at 5 min to 67.0% at 40 min. However, the inhibitor-treated parasites showed significantly less ingestion, with only 4.4% of parasites treated with 100 mM ammonium chloride ingesting a high number of human fragments after 40 min (Fig. 1A). Indeed, treatment with either ammonium chloride (Fig. 1B) or concanamycin A (Fig. 1C) significantly decreased amebic trogocytosis in a dose-dependent manner, as measured by the percentage of amebae that ingested a high number of human cell fragments. As expected, treatment with these inhibitors also decreased cell killing (Fig. 1D and E). Treatment with ammonium chloride alone did not directly cause human cell death (Fig. 1D). Human cells were not exposed to concanamycin A in this assay; therefore, we assessed the impact of incubation in medium alone and found that it also did not cause human cell death (Fig. 1E). Treatment with either of the inhibitors alone did not directly cause amebic death (see Fig. S2 in the supplemental material). Together, these data suggest a crucial role for amebic lysosomes in amebic trogocytosis.

10.1128/mBio.01187-17.2 FIG S2  Acidification inhibitors do not cause parasite death. Amebae pretreated with ammonium chloride for 1 h were incubated for 5, 20, or 40 min in medium containing ammonium chloride. Amebae pretreated with concanamycin or vehicle for 1 h were washed and incubated for 5, 20, or 40 min in medium without inhibitor or vehicle. Afterward all cells were stained with LIVE/DEAD violet on ice for 30 min and then fixed. Cell killing was analyzed using imaging flow cytometry. The percentage of dead amebae represents the percentage of amebae staining with LIVE/DEAD violet. Data were analyzed by one-way ANOVA with a multiple-comparison test using Prism 6. Download FIG S2, TIF file, 2.6 MB. Copyright © 2017 Gilmartin et al. 2017 Gilmartin et al. This content is distributed under the terms of the Creative Commons Attribution 4.0 International license.

### Acidification inhibitors do not impair initiation of amebic trogocytosis.

**FIG 2:**
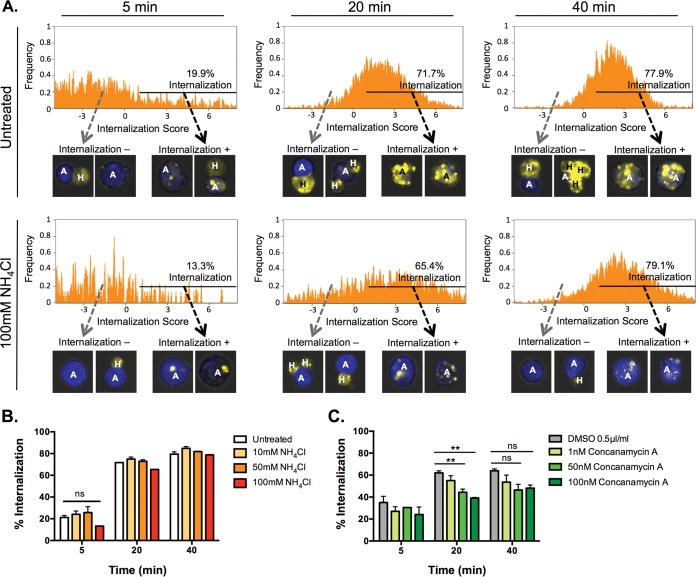
Acidification inhibitors do not inhibit initiation of amebic trogocytosis. Amebae were pretreated with inhibitor or vehicle for 1 h. (A and B) Ammonium chloride-treated amebae were incubated with Jurkat cells in medium containing ammonium chloride. (C) Amebae pretreated with concanamycin were washed and incubated with Jurkat cells in medium without inhibitor or vehicle. Amebic trogocytosis was analyzed using imaging flow cytometry. The percentage of internalization reflects the percentage of amebae that have ingested any host material. Means and standard deviations are for biological duplicates (10,000 events each). **, *P* ≤ 0.01; ns, not significant. Data were analyzed by one-way ANOVA.

To understand how amebic lysosomes participate in amebic trogocytosis, we first sought to determine when during the process of amebic trogocytosis inhibitor-treated parasites were impaired. Amebic trogocytosis entails a series of events that result in human cell death: attachment to the human cell, internalization of a human cell fragment, and then continued ingestion of multiple fragments. It is possible that the inhibitors impaired the ability of the parasites to attach to human cells and initiate trogocytosis. To determine whether the acidification inhibitors impaired initiation of trogocytosis, we assessed whether there was a difference in the ability of inhibitor-treated parasites to ingest any amount of human material compared to that of control parasites. We found that the percentage of control parasites that had internalized human material increased over time from 19.9% at 5 min to 77.9% at 40 min. Interestingly, similar percentages of parasites treated with 100 mM ammonium chloride (79.1%) had internalized human material by 40 min (Fig. 2A), indicating that a majority of amebae were able to ingest some human material by 40 min, regardless of ammonium chloride treatment. Overall, there was no difference in the percentage of inhibitor-treated parasites that internalized any human material and control parasites (Fig. 2B and C). These data indicate that the acidification inhibitors did not impair the initiation of trogocytosis. However, the inhibitors did decrease the percentage of parasites that ingested a high number of fragments (Fig. 1B and C), suggesting that the parasites were impaired in their ability to continue to trogocytose many fragments.

### Acidification inhibitors also decrease phagocytosis, but not fluid-phase endocytosis.

**FIG 3:**
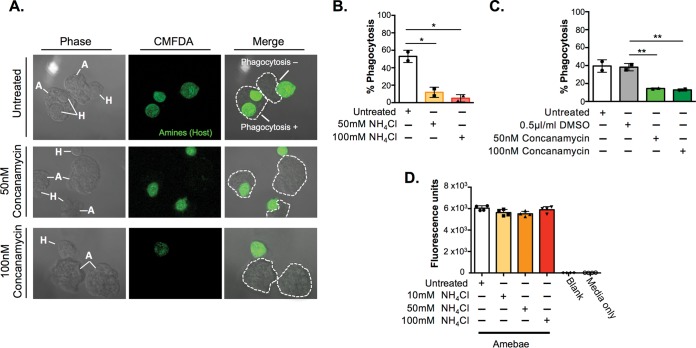
Acidification inhibitors decrease phagocytosis, but not fluid-phase endocytosis. Amebae were coincubated with heat-killed Jurkat cells for 10 min. After coincubation, all cells were fixed. Phagocytosis was analyzed using fluorescent confocal microscopy. (A) Confocal microscopy of unlabeled amebae and human Jurkat T cells prelabeled with CMFDA (amines are labeled green). (Top row) Amebae with one or more human cells inside their outer membrane were scored phagocytosis positive. (Middle and bottom rows) Representative images of amebae preincubated with concanamycin A. (B) Amebae pretreated with ammonium chloride for 1 h were incubated with heat-killed Jurkat cells for 10 min in medium containing ammonium chloride. (C) Amebae pretreated with concanamycin A for 1 h were washed and incubated with heat-killed Jurkat cells for 10 min in medium without inhibitor or vehicle. (D) Amebae pretreated with ammonium chloride for 1 h were incubated with RITC-dextran for 40 min in medium containing ammonium chloride, washed with PBS, and then permeabilized with 0.1% Triton X-100. Fluid-phase endocytosis was assessed using a fluorescence plate reader. Means and standard deviations are for biological duplicates (B and C) or technical quadruplicates (D). *, *P* ≤ 0.05; **, *P* ≤ 0.01. Data were analyzed by one-way ANOVA with Holm-Sidak’s multiple-comparison test using Prism 6.

Amebic trogocytosis (ingestion of cell fragments) shares many similarities to another endocytic process, phagocytosis (ingestion of whole cells). Both amebic trogocytosis and phagocytosis are receptor-dependent processes, requiring attachment to a target cell via the amebic Gal/GalNAc lectin (6, 8). These processes also require amebic actin rearrangement and PI3K signaling (6, 23, 24). Thus far, no pathways unique to trogocytosis have been discovered in any organism; therefore, we assessed the impact of the acidification inhibitors on phagocytosis. It has been demonstrated that *E. histolytica* will preferentially phagocytose (ingest whole) dead human cells and trogocytose (ingest fragments) live human cells (6). To measure the impact of acidification inhibitors on phagocytosis, we coincubated ammonium chloride-treated, concanamycin A-treated, and control parasites with CMFDA-labeled heat-killed human Jurkat T cells at 37°C (Fig. 3A). Amebae pretreated with ammonium chloride were coincubated with dead human cells in medium containing ammonium chloride, while amebae pretreated with concanamycin A were washed and coincubated with dead human cells in medium without inhibitor. Following coincubation, all cells were fixed and phagocytosis was assessed using confocal microscopy as previously described (23). Consistent with previous work (16), we found that treatment with either ammonium chloride or concanamycin A decreased phagocytosis (Fig. 3B and C).

In contrast to both amebic trogocytosis and phagocytosis, fluid-phase endocytosis is not receptor dependent and does not require large actin rearrangements. To determine whether the inhibition of lysosomal acidification impaired fluid-phase endocytosis, we incubated ammonium chloride-treated parasites with rhodamine B isothiocyanate (RITC)-dextran for 40 min in medium containing ammonium chloride and assessed ingestion of the dextran using a fluorescence plate reader. Intriguingly, we found that ammonium chloride treatment did not impact fluid-phase endocytosis (Fig. 3C). Taken together with our observations on amebic trogocytosis and phagocytosis, these data suggest that amebic lysosomes might play an important role in the rapid degradation of ingested fragments or recycling of membranes and receptors required for continued amebic trogocytosis.

## DISCUSSION

For decades it was unclear how *E. histolytica* killed human cells. Recently, we discovered that *E. histolytica* ingests fragments of live human cells through amebic trogocytosis, which leads to cell killing (6). However, the mechanism of amebic trogocytosis is not well understood. Previous work has demonstrated that attachment of the parasite to the host cell is mediated largely by the parasite surface receptor Gal/GalNAc lectin and that an active parasite cytoskeleton is required for the ingestion of cell fragments (6). Processes that are morphologically similar to amebic trogocytosis occur in other organisms, including humans and several eukaryotic parasites, but again the underlying mechanisms are poorly understood (9). In the present study, we demonstrated that interference with lysosomal acidification impairs amebic trogocytosis and cell killing, indicating a critical role for amebic lysosomes in both trogocytosis and cell killing.

It has been demonstrated that ingestion of host cell material via trogocytosis is the major mechanism of cell killing by *E. histolytica*. Previous work, which was done before the discovery of amebic trogocytosis, has shown that weak bases inhibit amebic killing of human cells (16). Interestingly, studies comparing *E. histolytica* with the less-pathogenic species *Entamoeba dispar* have noted that acidification of the phagosomes takes significantly longer in *E. dispar* and does not reach the same level of acidification, also indicating a possible role for lysosomes in the pathogenesis of amebiasis (18, 25). Indeed, chloroquine, a lysosomotropic weak base that impairs lysosomal acidification (26), has been used to successfully treat amebic liver abscesses (27). Our present findings shed new light on these observations by demonstrating that acid vesicle neutralization acts through the inhibition of trogocytosis and cell killing.

We have shown that interference with acidification does not impact the initiation of amebic trogocytosis, but rather impairs continued ingestion and cell killing. There was no difference in the abilities of untreated and acidification inhibitor-treated parasites to begin to ingest some human cell fragments, suggesting that the parasites were not simply impaired in their ability to attach to human cells (Fig. 2B and C). This is consistent with previous work showing that treatment of parasites with moderate amounts of ammonium chloride for as long as 48 h did not impact parasite attachment to mammalian cells or binding of colonic mucin (16, 28). However, the amount of human material ingested by the acidification inhibitor-treated parasites was drastically reduced, indicating that the parasites’ ability to continue ingesting human fragments was impaired. *E. histolytica* has been shown to acidify phagosomes within 2 min, suggesting that lysosomes are rapidly recruited and fuse with the phagosome (18). Our data are consistent with the hypothesis that the inhibitor-treated parasites fail in this rapid acidification step, resulting in slowed or blocked ingestion. Continued ingestion of multiple human fragments appears to be required to kill human cells (6): thus these parasites are also impaired in their ability to kill human cells, as we observed (Fig. 1D and E).

There are several possible roles that lysosomes might play in amebic trogocytosis: lysosomes might be required for efficient degradation of the ingested fragments, for rapid recycling of ingested receptors and membrane, or for the rapid formation of an acidified synapse at the site of ameba-host interaction. During amebic trogocytosis, parasites ingest numerous host cell fragments. In eukaryotes, lysosomes are crucial for the turnover of ingested material. Weak bases, such as ammonium chloride, raise the pH of amebic lysosomes, which would impair the function of pH-dependent lysosomal proteases (16). Previous work has also shown that concanamycin A-treated parasites failed to acidify their phagosomes to normal levels and were impaired in their ability to degrade phagocytosed *Leishmania* (18). It is possible that lysosomes are crucial for continued amebic trogocytosis and cell killing because they are required for efficient degradation of the ingested host fragments. Further study is needed to determine whether this is the case.

Our data show that interference with acidification blocks receptor-dependent processes—both amebic trogocytosis and phagocytosis—but does not impair a receptor-independent process, fluid-phase endocytosis. These findings are consistent with previous reports that treatment of parasites with ammonium chloride decreased phagocytosis of bacteria and, similarly, treatment with the V-ATPase inhibitor bafilomycin decreased ingestion of both bacteria and human red blood cells (24). Together, these data suggest that rapid recycling of membrane and receptors, facilitated by the amebic lysosomes, may be required for continued amebic trogocytosis and, thus, cell killing.

It has also been suggested that amebic lysosomes may form an acidified synapse at the site of host cell attachment similar to the synapse created by mammalian osteoclasts, which secrete lysosomes into an acidified synapse during bone matrix degradation (29). Live confocal microscopy and electron microscopy have both shown a massive accumulation of actin and exclusion of vesicles at the site of active host fragment ingestion, making this hypothesis less likely (6). However, it is still possible that amebic lysosomes fuse at the ameba-host cell interface after attachment, but before active ingestion begins. Further study is needed to examine whether such a synapse is formed.

We acknowledge that, in addition to impairing lysosomal function, the acidification inhibitors used in this study are likely to impact other vesicles in the endocytic pathway. In this study, we have used concanamycin A, which specifically inhibits V-ATPases to block vesicle acidification. Studies in mammalian systems have shown that during endosome/phagosome maturation, vesicles accumulate V-ATPase complexes on their membranes and become progressively more acidic. V-ATPases are also involved in regulating the pH of sorting and recycling endosomes (reviewed in reference 30). The impact of concanamycin A is likely to be most profound on the lysosomes, which have been shown in mammalian systems to have the greatest accumulation of V-ATPases, the lowest pH of vesicles in the cell, and a strong dependence on acidic pH for their function. Similarly, ammonium chloride is a membrane-permeable weak base and therefore may impact the acidification of other vesicles in the amebic endocytic pathway. However, ammonium chloride has been described as a lysosomotropic agent for its propensity to accumulate in lysosomes (31). Like concanamycin A, ammonium chloride is likely to have the greatest impact on the lysosomes. In *Entamoeba histolytica*, it has been shown that exposure to as little as 10 mM ammonium chloride for 20 min increased the acid vesicle pH to 6.11 (16). While the endocytic pathway is an active area of study, we currently lack a well-defined set of markers to distinguish different vesicles within the endocytic pathway. Future discovery of such markers may allow for a further examination of the impact of these acidification inhibitors on specific vesicles within the endocytic pathway.

Tissue destruction is the hallmark of invasive *E. histolytica* infection, and parasite cytotoxic activity is likely to drive tissue destruction. It has recently been discovered that human cell death occurs as a result of amebic trogocytosis. This work sheds new light on an observation, first made 30 years ago, that weak bases inhibit amebic killing of human cells, by demonstrating that acid vesicle neutralization acts through the inhibition of trogocytosis. This work will contribute to a better understanding of the nature of trogocytosis as a fundamental biological process.

## MATERIALS AND METHODS

### Cell culture.

Amebic trophozoites (HM1:IMSS) were cultured axenically at 35°C in TYI-S-33 as previously described (23). Trophozoites were harvested during log-phase growth by centrifugation at 200 × *g* for 5 min at room temperature, followed by resuspension in M199S (medium 199 [Gibco] without phenol red and supplemented with 5.7 mM cysteine [Sigma], 0.5% bovine serum albumin [Gemini], and 25 mM HEPES [Sigma] at pH 6.8) (6).

Human Jurkat cells (clone E6-1; ATCC) were grown at 37°C in RPMI 1640 (Gibco) supplemented with 10% fetal bovine serum (Gibco), 10 mM HEPES (Gibco), and 1 mM sodium pyruvate (Gibco). Jurkat cultures were collected and enriched for viable cells as previously described (6).

### Amebic trogocytosis and cell killing assay.

Amebic trogocytosis by amebae was measured using imaging flow cytometry as described previously with some modifications (6). Briefly, amebae were labeled with 200 nM CellTracker Green 5-chloromethylfluorescein diacetate (CMFDA; Invitrogen) in M199S for 10 min at 37°C and then washed twice with M199S. Following CMFDA labeling, amebae were treated with pharmacological inhibitors or vehicle control for 1 h at 37°C. Amebae were treated either with ammonium chloride (Sigma) at 10, 50, or 100 mM or with M199S as a control, or amebae were treated with concanamycin A (Sigma) at 10, 50, or 100 nM or an equal volume of dimethyl sulfoxide (DMSO) (Molecular Probes) as a vehicle control. Jurkat cells were labeled with 5 µM DiD (Assay Biotech) in M199S for 5 min at 37C, followed by 10 min at 4C, and then washed twice with M199S. Amebae pretreated with ammonium chloride were coincubated with labeled Jurkat cells at a ratio of 1:5, in biological duplicate at 37°C for 5, 20, or 40 min in M199S containing ammonium chloride. Amebae pretreated with concanamycin A or DMSO were washed twice with M199S and then coincubated with labeled Jurkat cells in biological duplicates at 37°C for 5, 20, or 40 min in M199S. At the end of each time point, samples were immediately placed on ice and labeled with LIVE/DEAD fixable violet (Invitrogen) at 1.6 µl/ml for 30 min in the dark. The cells were then fixed with 4% paraformaldehyde in phosphate-buffered saline (PBS) for 30 min at room temperature in the dark. Flow cytometry was performed using ImageStreamX Mark II (EMD Millipore). A total of 10,000 events were collected for each sample, and data were analyzed using IDEAS software (EMD Millipore).

### Phagocytosis assay.

Jurkat cells were collected, washed with M199S, and labeled with 5 µM CMFDA (Invitrogen) for 15 min at 37°C as previously described (6). Cells were then washed with M199S, and necrotic cell death was induced by incubation of Jurkat cells at 55°C for 15 min (23). Amebae were collected, washed with M199S, and treated with pharmacological inhibitors for 1 h at 37°C. Amebae were treated either with ammonium chloride (Sigma) at 10, 50, or 100 mM or with M199S as a control; alternatively, amebae were treated with concanamycin A (Sigma) at 10, 50, or 100 nM or an equal volume of DMSO as a vehicle control. Amebae pretreated with ammonium chloride were coincubated with heat-killed Jurkat cells at a ratio of 1:5 in M199S containing ammonium chloride for 10 min at 37°C. Amebae pretreated with concanamycin A or DMSO were washed with M199S and then coincubated with labeled Jurkat cells in M199S for 10 min at 37°C. After coincubation, all samples were fixed with 4% paraformaldehyde in PBS for 30 min at room temperature. All samples were washed with PBS, mounted with Vectashield H-1000 antifading agent, and imaged using Zeiss LSM software on a Zeiss LSM700 inverted confocal microscope equipped with a 63× apochromatic oil objective (6). Fields containing two or more amebae were imaged, and amebae containing one or more human cells were scored phagocytosis positive. Results were expressed as percentage of phagocytosis, which is the number of amebae containing at least 1 Jurkat cell per slide divided by the total number of amebae imaged per slide.

### Fluid-phase endocytosis assay.

The fluid-phase endocytosis assay was performed as described previously, with some modifications (32). Briefly, amebae were collected, washed with M199S, and then treated with pharmacological inhibitors for 1 h at 37°C. Approximately 2.5 × 10^5^ amebae were incubated with 2 mg/ml rhodamine B isothiocyanate (RITC)-dextran (molecular weight, 10,000; Invitrogen) for 40 min at 37°C. Amebae were then washed extensively with PBS and permeabilized with 0.1% Triton X-100. Samples were plated in quadruplicate on a 96-well black solid plate (Corning). Fluid-phase endocytosis assessed by measuring fluorescence intensity after excitation at 570 nm using a fluorescence plate reader (BioTek).

### Statistical analysis.

Data were analyzed using Prism 6 (GraphPad Software, Inc.).
