# Supplementary figures and images for: Inhibition of Amebic Lysosomal Acidification Blocks Amebic Trogocytosis and Cell Killing
Source: mBio. 2017 Aug 29;8(4):e01187-17. doi: 10.1128/mBio.01187-17 (PMC5574710; doi:10.1128/mBio.01187-17)

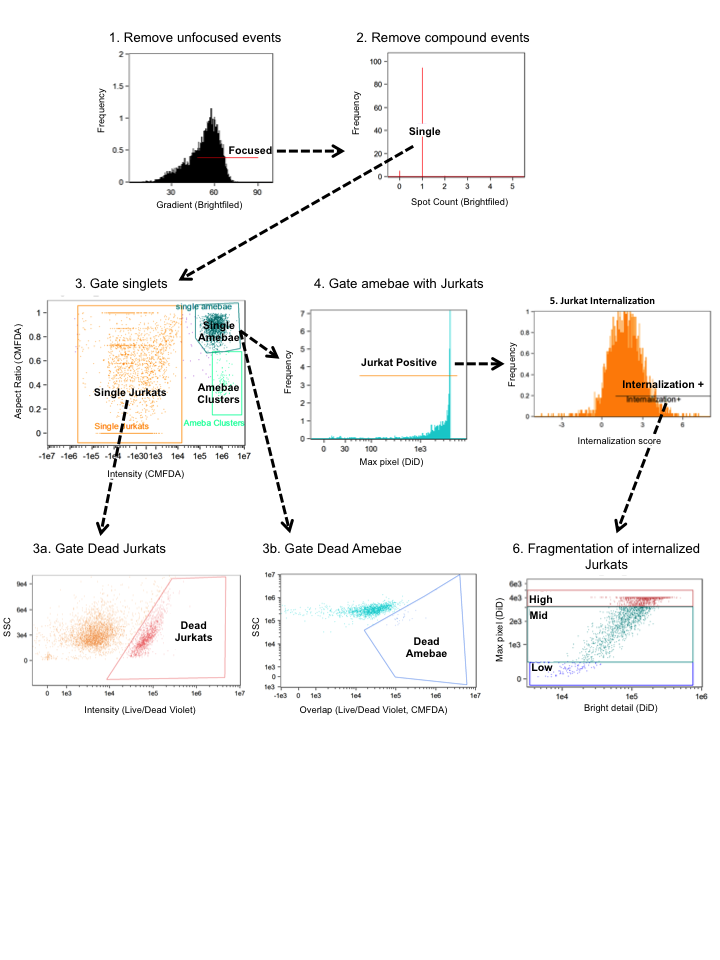

Supplement: FIG S1 [file mbo004173457sf1.tif]

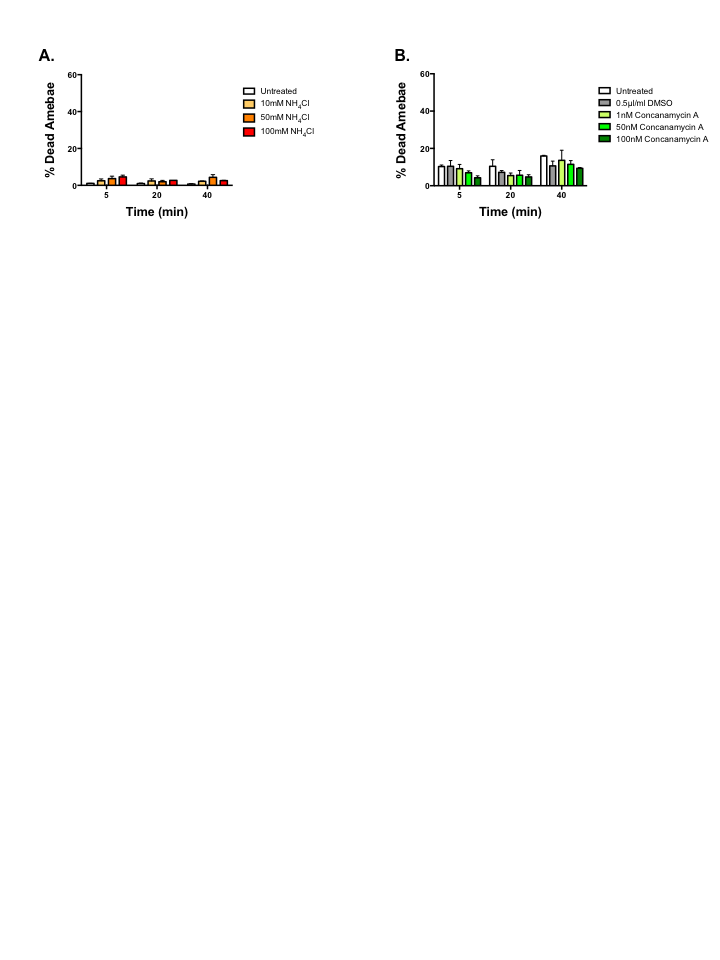

Supplement: FIG S2 [file mbo004173457sf2.tif]
